# Validation of a rapid on-farm culture system for group classification of clinical mastitis-causing pathogens

**DOI:** 10.3168/jdsc.2024-0738

**Published:** 2025-04-11

**Authors:** Fernando J. Guardado Servellon, David L. Renaud, Bruno Joaquin Paredes Osorio, Kelsey L. Spence, Trevor J. DeVries, Rita Couto Serrenho

**Affiliations:** 1 Population Medicine, University of Guelph, Guelph, ON N1G 2W1, Canada; 2 Eldale Veterinary Clinic, Elmira, ON N3B 1N3, Canada; 3 Animal Biosciences, University of Guelph, Guelph, ON N1G 2W1, Canada

## Abstract

The objective of this diagnostic accuracy study was to compare the use of a rapid tube test system (MastDecide [MD]; Quidee GmbH, Homberg, Germany) to aerobic milk culture for group classification of mastitis pathogens in dairy cattle. A total of 204 milk samples from cows with clinical mastitis (CM) were collected across 60 dairy herds in Ontario, Canada. Samples were collected by dairy producers and their staff and transported refrigerated to the University of Guelph Animal Health Laboratory (Guelph, ON, Canada) on the day of collection. Each sample was tested via MD (gram-positive, gram-negative, or “no growth”) and via aerobic milk culture followed by MALDI-TOF (reference method [RM]). The MD and RM results were interpreted at 14 and 24 h after incubation, respectively. An additional assessment was performed regarding the accuracy of intramammary antibiotic treatment decisions (gram-positive: to treat; gram-negative and “no growth” samples: not to treat). Test performance characteristics (overall accuracy, sensitivity [Se], specificity [Sp], and positive [PPV] and negative predictive value [NPV]) as well as Cohen's kappa coefficient (κ) were calculated. The RM results were classified as gram-positive (n = 107, 53%), gram-negative (n = 20, 10%), “no growth” (n = 51, 25%), other pathogens (n = 21, 10%), and mixed growth (gram-positive and gram-negative; n = 5, 3%). The Se of the MD test was 58% (95% CI: 47%–67%), 40% (19%–64%), and 61% (50%–72%) for gram-positive, gram-negative, and “no growth,” respectively. The Sp of the MD test was 73% (63%–81%), 84% (78%–89%), and 75% (66%–82%) for gram-positive, gram-negative, and “no growth,” respectively. The PPV of the MD test was 68% (57%–78%), 22% (10%–39%), and 62% (50%–73%) for gram-positive, gram-negative, and “no growth,” respectively; and the NPV was 63% (54%–72%), 93% (87%–96%), and 74% (65%–82%) for gram-positive, gram-negative, and “no growth,” respectively. The overall test accuracy for Gram stain classification analysis was 57%, with a κ of 0.30. Regarding intramammary antibiotic treatment, the MD test yielded a correct decision relative to the RM for 65% of the cases. Using this on-farm test as a primary guide in CM treatment decisions should be approached with caution, as the risk of false negatives may affect the ability to effectively treat CM cases.

## Highlights

• The test evaluated is prone to false negatives.
• The test evaluated has a lower analytical sensitivity than the reference method.
• The test is suboptimal for the classification of mastitis pathogens from CM samples.

## Graphical Abstract

**Figure:**
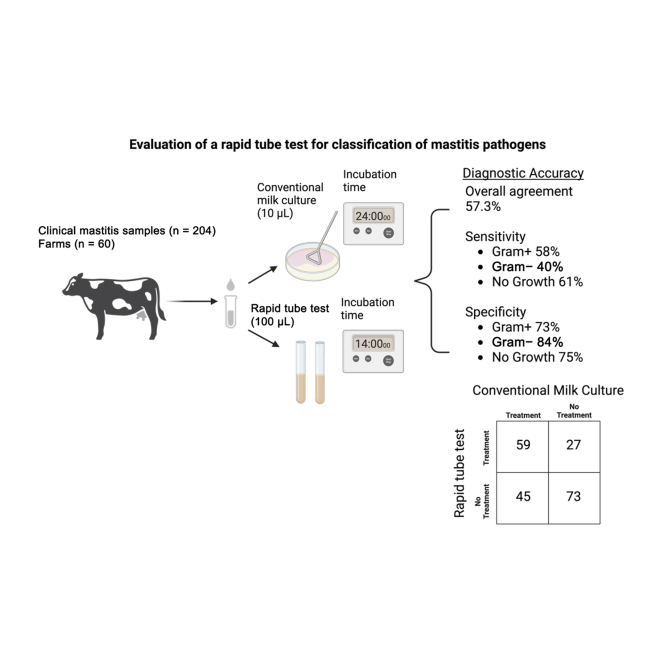
**Summary:** The objective of this test accuracy study was to evaluate a rapid test tube milk culture for classification of Gram group of clinical mastitis (CM) pathogens as compared with conventional milk culture with identification using matrix-assisted laser desorption/ionization time-of-flight. An accurate method with a shorter turnaround time than conventional milk culture may aid in on-farm implementation of selective therapy protocols for CM. Overall, the performance of the test evaluated was suboptimal for the classification of mastitis pathogens from CM samples.

The reduction and judicious use of antimicrobials are top priorities for the dairy industry, driven in part by consumer concerns about antimicrobial use (**AMU**) in dairy animals and its perceived threat to human health (Wemette et al., 2021). Decreased AMU has been associated with a reduction in antimicrobial resistance in food animal species (Tang et al., 2017), highlighting the importance of responsible AMU practices. Approximately 66% of AMU on Canadian dairy farms is attributed to clinical mastitis (**CM**) treatment and dry-off therapy (Warder et al., 2023). A selective antimicrobial therapy approach for CM can assist producers in reducing AMU without compromising animal health (Vasquez et al., 2017). In cases of nonsevere CM, selective treatment can achieve comparable outcomes to blanket treatment with no statistical differences in cure rates, SCC, milk yield, or risk of recurrence and culling due to mastitis found in many clinical trials (Bates et al., 2020, Beinhauerova et al., 2023; Griffioen et al., 2021; Borchardt and Heuwieser, 2022). Selective CM treatment also offers economic advantages, for example by reducing treatment costs and minimizing milk withdrawal periods (Pinzón-Sánchez et al., 2011).

Selective treatment of CM cases can be guided by determining the Gram stain classification of the causative bacteria (Lago and Godden, 2018). Bacteria are classified as gram-positive or gram-negative using selective media, which support the growth of one group while inhibiting the other, or by applying a Gram stain to the isolates grown on nonselective media (Suzuki and Isobe, 2025). Mastitis caused by gram-positive bacteria, such as NAS species and *Streptococcus*, generally have greater cure rates when treated with antimicrobials compared with untreated cases (Apparao et al., 2009). In most mastitis cases caused by gram-negative bacteria, antimicrobial therapy does not improve outcomes compared with no treatment (Fuenzalida and Ruegg, 2019). Similarly, mastitis caused by *Prototheca* spp., yeast, and *Mycoplasma* spp., as well as cases represented by samples with no bacterial growth, do not benefit from antimicrobial therapy (Gelgie et al., 2024). Thus, tests that classify CM-causing bacteria by Gram stain classification can be used to guide treatment decisions (Malcata et al., 2020).

Milk bacteriological culture is considered the reference method for mastitis-causing pathogen identification (Dohoo et al., 2011). However, the turnaround time required for this test is ≥24 h, making it less practical for producers to implement a CM targeted therapy strategy based on its result (Malcata et al., 2020). Different on-farm diagnostic (**OFD**) systems were developed to overcome some of the shortcomings of aerobic milk culture (McCarron et al., 2009b). According to Kosack et al. (2017), an ideal diagnostic test should meet the ASSURED criteria: affordable, sensitive, specific, user-friendly, rapid and robust, equipment-free, and deliverable to end-users. Although agar-based tests are practical to conduct on-farm, they require considerable user training before implementation (McCarron et al., 2009b). User-friendly OFD with simplified interpretation show promise in increasing the adoption of selective treatment of CM on dairy farms (Jones et al., 2019). A simple OFD system based in a rapid tube test that provides results within 14 h is commercially available to assist producers in making CM antimicrobial treatment decisions (Leimbach and Krömker, 2018). Leimbach and Krömker (2018) evaluated the test performance in a laboratory environment, conducting both aerobic milk culture and the rapid tube test on the same sample. Overall, the authors reported a range in sensitivity (**Se**) of 71% to 84%, specificity (**Sp**) of 83% to 94%, positive predictive value (**PPV**) of 42% to 92%, and negative predictive value (**NPV**) of 83% to 95%, for gram-positive, coliform bacteria, and “no growth.” The objective of this cross-sectional diagnostic accuracy study was to evaluate the performance of a rapid tube test system (MastDecide [**MD**]; Quidee GmbH, Homberg, Germany) compared to aerobic milk culture (reference method [**RM**]) under conditions representative of a farm environment. We hypothesized that the MD performance would align with previously reported results from a laboratory setting (Leimbach and Krömker, 2018).

This study is reported using the Standards for Reporting of Diagnostic Accuracy Studies guidelines (Cohen et al., 2016). Milk samples were collected between May and July 2024, totaling 204 samples from 60 dairy farms in southwestern Ontario, Canada. Farms were selected through convenience sampling, consisting of clientele from 3 local veterinary clinics. The definition of a CM case (changes in foremilk such blood, flakes, clots, or a watery appearance, including mild, moderate, or severe cases; Morin et al., 1998) and how to aseptically collect milk samples were discussed and agreed upon with the farmers by the herd veterinarians at the beginning of the study. Composite milk samples were considered if producers observed more than one quarter with abnormal milk (abnormal milk appearance in quarters adjacent to the infected quarter could cause challenges to distinguish the affected quarter; Paixão et al., 2017). Composite or individual quarter milk samples of ∼20 mL were collected in a sterile vial by farm staff or their veterinarian. Samples were kept refrigerated and transported to the University of Guelph (Guelph, ON, Canada) on the day of collection.

The MD test is a point-of-care tube system that uses liquid growth media with colorimetric properties to classify samples as gram-positive, gram-negative, or “no growth.” The MD test is unable to detect *Prototheca* spp., *Corynebacterium* spp., *Bacillus* spp., *Pseudomonas* spp., *Mycoplasma* spp., *Trueperella pyogenes,* or yeast growth (Leimbach and Krömker, 2018). The test consists of 2 colorimetric test tubes; one shows discoloration with gram-positive growth, and the other discolors with either growth of gram-positive or gram-negative pathogens. On the same day the sample was received, it was gently agitated and 200 µL (100 µL for each of the MD test tubes) were removed using a sterile micropipette to conduct the MD assay. After adding the inoculum to the 2 test tubes, they were gently agitated and incubated for 14 h in an egg incubator (Little Giant 11300 Deluxe Digital Incubator; Miller Manufacturing, Glencoe, MN) at 35°C. The samples were read by a single observer after 14 h (incubation time suggested by the manufacturer to maximize Se). Before the beginning of the study, the observer became familiarized with the manufacturer's instructional manual by practicing reading mastitic samples including all 3 possible outcomes. The MD test results were interpreted as follows: gram-positive (indicated by discoloration of both tubes), gram-negative (one discolored tube), and “no growth” (no discolored tubes).

The remaining milk was kept refrigerated, and it was submitted within 30 min for the RM at the University of Guelph Animal Health Laboratory (Guelph, ON, Canada). For the RM, 10 µL of milk was streaked on both Columbia blood agar (**BA**) and MacConkey agar (**MAC**) plates according to the Animal Health Laboratory standard operating procedure. The agar plates were incubated overnight at 35°C. The BA was incubated in the presence of 5% CO_2_, and the MAC were incubated in the atmospheric air. The BA and MAC plates were assessed at 24 and 48 h of incubation for the presence of bacterial growth. Samples were considered positive if growth (at least one colony type with >100 cfu/mL or >200 cfu/mL for NAS; National Mastitis Council, 2017) was observed within 48 h of incubation and considered as “no growth” (negative samples) if no bacterial growth observed. Samples with 3 or more colony types would be classified as contaminated (Dean et al., 2022). Final bacterial identification was done by MALDI-TOF Biotyper sirius System (reference library version 4.1.100; species identification: log score of ≥2.0; genus identification: log score from 1.7 to 1.999; Bruker Daltonics Inc., Bremen, Germany).

The minimum required sample size to evaluate the Se and Sp of the MD as compared with the RM was calculated as previously described by Buderer (1996). We accounted for a 30% prevalence of CM. We calculated the sample size using a Se of 80% and a clinically acceptable width of the 95% CI for Se of 10%, requiring a minimum of 205 samples. Multiple farms were included to comprise different causal pathogen distributions, as well as to obtain enough CM cases during the study period. Data were recorded in Microsoft Excel (version 16.88; Microsoft Corp., Redmond, WA) and analyzed using R version 4.4.1 (R Core Team, 2024). Calculation of test performance characteristics was performed with the epiR package (Stevenson, 2024) and included overall accuracy, Se, Sp, PPV, NPV, as well as Cohen's kappa coefficient of agreement between tests (κ) as described in Ferreira et al. (2018). The κ (agreement beyond chance) was classified as “none to slight” (0.01–0.20), “fair” (0.21–0.40), “moderate” (0.41– 0.60), “substantial” (0.61–0.80), and “almost perfect” (0.81–1.00; McHugh, 2012).

We analyzed the data (1) considering “no growth,” gram-positive, or gram-negative, and (2) considering treatment decision (“to treat” if gram-positive, vs. “not to treat” if gram-negative or “no growth”). We analyzed the dataset using multiple approaches, considering different population subsets (all samples [n = 199], without composite samples [n = 134], only pathogens that the MD test could grow [n = 178], and samples other than *Staphylococcus aureus* [n = 165]). Because all the approaches had similar results with no substantial changes in test characteristics, we reported results of the scenario with the largest sample size. Due to the need to group samples according to a specific Gram stain classification, samples with mixed growth (gram-positive and negative bacteria growth; n = 5) were excluded from the Gram stain analyses. However, the mixed growth samples were considered in the intramammary antibiotic treatment decision analysis. Samples that grew undetectable pathogens by the MD on the RM test were considered as “no growth.”

A total of 204 samples from CM cases from 60 dairy farms were received. The RM results yielded 53% (107/204) gram-positive, 10% (20/204) gram-negative, 25% (51/204) “no growth,” and 10% (21/204) nondetectable pathogens by the MD (Table 1). Five samples (2%) were excluded from the Gram stain classification analyses due to mixed growth (gram-positive and gram-negative bacteria on the same sample). In total, 199 milk samples were considered for the Gram stain classification (134 individual quarter samples and 65 composite samples; Table 1), and 204 milk samples were considered for treatment decision analysis. The RM classified 148/199 samples (74%) as having IMI.

**Table 1:** Aerobic milk culture and group classification of 204 clinical mastitis (CM) samples

Group classification	Microorganism	CM samples, n (%)
Gram-positive (52.5%)	*Staphylococcus aureus*	34 (16.7)
	*Streptococcus uberis*	24 (11.8)
	*Streptococcus dysgalactiae*	8 (3.9)
	*Enterococcus* spp.	6 (3)
	Non-*aureus* staphylococci	21 (10.3)
	Mixed gram-positive	14 (6.9)
Gram-negative (9.8%)	*Escherichia coli*	8 (3.9)
	*Pasteurella* spp.	7 (3.4)
	*Klebsiella* spp.	2 (1)
	*Serratia* spp.	1 (0.5)
	Mixed gram-negative	2 (1)
Mixed growth (2.4%)	Gram-positive and gram-negative^1^	5 (2.4)
Other pathogens (10.3%)^2^	*Corynebacterium* spp.	10 (4.9)
	*Prototheca* spp.	2 (1)
	*Trueperella pyogenes*	3 (1.5)
	Yeast	6 (2.9)
No growth (25.0%)	—	51 (25)
Contaminated^3^	—	0 (0)

1 The following pairings were excluded from the test performance analysis: *E. coli*,*Staph. aureus*;*Staphylococcus chromogenes*,*Serratia* spp.; *E. coli*,*Staph. aureus*, *Klebsiella* spp., *Streptococcus dysgalactiae*; *Streptococcus uberis*,*Corynebacterium* spp.

2 Because the MD test is unable to detect some pathogens (*Prototheca* spp., *Corynebacterium* spp., *Bacillus* spp., *Pseudomonas* spp., *Mycoplasma* spp., *Trueperella pyogenes*, or yeast), for test performance analysis, these were considered “no growth.”

3 Contamination was defined as the growth of 3 distinct bacterial species from the same sample.

The MD test classified 84/199 of the samples (42%) as gram-positive, 36/199 (18%) as gram-negative, and 79/199 (40%) as “no growth.” The MD classified 120/199 samples (60%) as having IMI. The κ for the Gram stain classification was fair (0.33 [0.22–0.44]). The test performance characteristics of the MD for gram-positive, gram-negative, and “no growth” classification are presented in Table 2. Without the composite samples included (n = 146), the MD showed similar performance with an Se of 56% (95% CI: 43%–69%), 58% (45%–70%), and 50% (26%–74%), and an Sp of 70% (58%–79%), 76% (65%–84%) and 85% (78%–91%) for gram-positive, gram-negative, and no growth, respectively.

**Table 2:** Test performance characteristics and agreement of a rapid tube system for on-farm culture of mastitis (MastDecide [MD]; Quidee GmbH, Homberg, Germany) assessed on 199 fresh clinical mastitis samples when compared with aerobic milk culture (reference method); results are presented with 95% CI in parentheses

Test characteristics	Gram-positive (n = 107)	Gram-negative (n = 20)	No growth^1^ (n = 72)
Sensitivity, %	58 (47–67)	40 (19–64)	61 (50–72)
Specificity, %	73 (63–81)	84 (78–89)	75 (66–82)
Positive predictive value, %	68 (57–78)	22 (10–39)	62 (50–73)
Negative predictive value, %	63 (54–72)	93 (87–96)	74 (65–82)
Overall accuracy, %	57		

1 Because the MD test is unable to detect some pathogens (*Prototheca* spp., *Corynebacterium* spp., *Bacillus* spp., *Pseudomonas* spp., *Mycoplasma* spp., *Trueperella pyogenes*, or yeast), for test performance analysis, these were considered “no growth.”

Compared with blanket therapy, a selective CM therapy strategy using the RM and MD would represent a 47% and 58% reduction in AMU, respectively. The Se, Sp, PPV, and NPV of the MD test for antimicrobial treatment were 57% (95% CI: 47%–66%), 73% (63%–81%), 69% (58%–78%), and 62% (52%–71%), respectively. The κ for the treatment decision was fair (0.29 [0.16–0.43]). If treatment decisions had been based on the MD test, 35% (n = 72) of the samples would have been misclassified (overall accuracy: 65%). Among the misclassified samples, 13% (n = 27) would have received unnecessary treatment, whereas 22% (n = 45) would have remained untreated despite potentially benefiting from it.

Milk bacteriological culture is widely accepted as the method of choice for diagnosis of IMI (Dohoo et al., 2011) and commonly used for clinical validity assessments of new OFD tests. In this test accuracy study conducted under farm-like conditions, the rapid OFD test demonstrated suboptimal Se (<70%), a moderate Sp (72%–86%), and a “fair” agreement in classifying pathogens by Gram stain classification versus bacteriological culture. These characteristics led to limited accuracy in distinguishing cases that required antimicrobial treatment from those that did not. Although the evaluated OFD test meets some of the ASSURED criteria (Kosack et al., 2017), namely in its speed of result delivery and user-friendliness), its performance characteristics led to substantial misclassification of CM samples, which may limit its effectiveness as a diagnostic tool for making antimicrobial treatment decisions.

The results of our study contrast with the previous validation study (n = 251 CM samples from 11 farms) conducted in a laboratory setting (100 mL of milk for the MD test and 100 mL for the microbiological culture; Leimbach and Krömker, 2018). In our study, the overall accuracy was 57%, which is lower agreement than the 79% previously reported by Leimbach and Krömker (2018). The authors reported an overall Se of 81% (75%–87%) and Sp of 71% (62%–80%) after reading results for the MD at 14 h after incubation and comparing it to the RM after 48 h of incubation. However, any direct comparison between performance values should be approached with caution due to differences in the methods used between the 2 studies, the most notable being the RM threshold for bacterial growth detection. In our study, the reference laboratory used a minimum threshold of 100 cfu/mL to consider bacteria growth (recommended for all bacterial pathogens except NAS, for which the detection limit is 200 cfu/mL; Dohoo et al., 2011), which contrasts with the 300 cfu/mL threshold for the RM in Leimbach and Krömker (2018).

In our study, the most common misclassification for true gram-positive and gram-negative growth was “no growth” by the MD test. This was likely due to a lower analytical Se of the MD test for both pathogen classes when compared with the RM. Although the Se and Sp of a test remain independent of pathogen prevalence in the assessed population, the PPV and NPV vary based on pathogen distribution (Rossi, 2021). Both PPV and NPV are crucial for interpreting test results in a real-world setting, as they reflect the probability that a positive or negative test result correctly identifies infection status. The PPV and NPV are prevalence-dependent, influencing the effectiveness of diagnostic and treatment decisions based on the distribution of pathogen in a given sample (Rossi, 2021). Although not expected, the small proportion of gram-negative samples in our population might have reduced the PPV for that pathogen class.

We included composite samples and those from individual quarters. Composite samples (n = 65), due to potential dilution effect, might have decreased analytic sensitivity for the isolation of pathogens (Reyher and Dohoo, 2011). We expect that in this case, the misclassification would be nondifferential, as that affects both the RM and the MD testing similarly. To further assess this potential bias, we evaluated the data with and without composite samples, and the performance results were comparable. A further limitation is that the incubation of MD and RM samples was not done in parallel, which may have introduced inconsistencies in bacterial growth or contamination, potentially leading to discrepancies in the results.

The test's inability to identify the specific causative pathogen can limit treatment decisions, as some gram-negative pathogens may respond to intramammary antimicrobial treatment, whereas some gram-positive pathogens may not (a limitation that applies to all tests that only classify bacteria by Gram stain). For example, cure rates can vary among gram-positive pathogens, with some IMM infections, such as *Staphylococcus aureus* or chronic cases of *Streptococcus uberis*, exhibiting lower treatment success (Apparao et al., 2009; Samson et al., 2016). Additionally, some strains of *Klebsiella* spp., a gram-negative pathogen, might respond favorably to antimicrobial therapy (Fuenzalida and Ruegg, 2019).

The test evaluated compares favorably in terms of convenience and turnaround time to other OFD systems. The MD requires an incubation time of 14 h which is less than the 24 h incubation period required for both the 3M Petrifilm (3M Microbiology, St. Paul, MN) and the Minnesota Easy Culture System II Biplate (University of Minnesota Laboratory for Udder Health, St. Paul, MN). The colorimetric property of the MD makes interpretation of results accessible for individuals without training, as opposed to agar-based methods (McCarron et al., 2009a). However, the Se for detection of gram-positive bacteria for the MD in the current study (58%) is lower than that of the 3M Petrifilm (94%) and the Minnesota Easy Culture System II Biplate (98%; McCarron et al., 2009b). Other colorimetric agar tests have been evaluated for use on-farm (Sipka et al., 2021; Griffioen et al., 2021). The AccuMast (FERA Diagnostics and Biologicals, College Station, TX) has shown a Se of 98% and Sp of 85% (Ferreira et al., 2018) which is superior to the performance of the MD in our study. The CHROMagar (CHROMagar, Paris, France) demonstrated a Se between 47% and 67% and Sp between 65% and 85%, similar to the values obtained for the MD in our study (Griffioen et al., 2021). In general, the MD results from our study demonstrated inferior test performance characteristics when compared with other commercially available OFD.

Overall, the performance of the test evaluated is suboptimal for the classification of mastitis pathogens at the gram level from CM samples. The low Se of the MD makes the test more prone to false negative misclassification (negative samples that are positive to the RM) when compared with the RM. Using this OFD as a primary guide in CM treatment decisions should be approached with caution, as the risk of false negatives may potentially compromise the ability to effectively treat CM cases.
